# Vincent’s Angina among the Troops in France

**Published:** 1919

**Authors:** 


***Vincent's Angina among the Troops in France.*** By R. J. C. Bouty. The *Laryngoscope*, April 1918.

In peace times this disease forms about two to three per cent. of all cases of throat trouble in the French Army, while recent statistics from a British military hospital in France show the proportion to be as high as twenty-three per cent.

From a study of cases under his own observation, Bouty found that this disease is characterized by the formation of ulcers on the buccal and pharyngeal mucous membrane, either superficial or deep, covered by a pseudomembrane. The most common site is the tonsil.

Microscopically, two varieties of organism are found: the bacillus fusiformis and Vincent's spirochete. Two forms of Vincent's angina are met with — (1) the pseudo-membranous, resembling diphtheria in appearance; and (2) the ulcerative form. In the first we find abundant bacilli mixed with cocci, and in the second, associated with the bacillus, a Gram-negative, mobile, flagellated spirillum. A coexisting streptococcal infection is not uncommon, and may give rise to serious complications.

The Ziehl-Nielson method is all that is required, though the silver method demonstrates the spirochete more clearly. Injected into animals the organisms are non-pathogenic. Injection of impure cultures causes abscesses in which the spirochete is abundant.

In the treatment of this condition Vincent states that mercurial treatment aggravates the condition when the patient is not a syphilitic. Arsenic and a mixture of sodium salicylate and potassium chlorate have been used, and tonics locally. Mercury perchloride and glycerin (1 in 500), silver nitrate, methylene blue, neosalvarsan, solution or powder applied daily for three days, and frequent gargles of hot hydrogen peroxide have been recommended.

The treatment which Vincent himself finds most effective is a thorough painting with tincture of iodine, 6 o/o after the membrane has been thoroughly removed.

In some cases a dose of antidiphtherial serum has given much relief. The importance of treating the unaffected side of the throat must be borne in mind to prevent occurrence on that side. Salvarsan intravenously (one dose) has been said to modify this condition and to prevent occurrence.

Vincent's angina is to be diagnosed from diphtheria, all types of tonsilitis, syphilitic ulceration, primary and tertiary, and scarlet fever. In every case a culture should be made to establish the absence of Loeffler's bacillus. The Wassermann reaction in this condition is negative. Certain skin conditions, such as erythemata and bullous symptoms, have been reported, but are probably due to coincident streptococcal infection.

